# Endless forms of sexual selection

**DOI:** 10.7717/peerj.7988

**Published:** 2019-11-05

**Authors:** Willow R. Lindsay, Staffan Andersson, Badreddine Bererhi, Jacob Höglund, Arild Johnsen, Charlotta Kvarnemo, Erica H. Leder, Jan T. Lifjeld, Calum E. Ninnes, Mats Olsson, Geoff A. Parker, Tommaso Pizzari, Anna Qvarnström, Rebecca J. Safran, Ola Svensson, Scott V. Edwards

**Affiliations:** 1 Department of Biological and Environmental Sciences, University of Gothenburg, Göteborg, Sweden; 2 Department of Ecology and Genetics, Uppsala University, Uppsala, Sweden; 3 Natural History Museum, University of Oslo, Oslo, Norway; 4 Department of Entomology and Nematology, University of Florida, Gainesville, FL, United States of America; 5 Institute of Integrative Biology, University of Liverpool, Liverpool, United Kingdom; 6 Department of Zoology, Edward Grey Institute, University of Oxford, Oxford, United Kingdom; 7 Department of Ecology and Evolutionary Biology, University of Colorado, Boulder, CO, United States of America; 8 School of Natural Sciences, Technology and Environmental Studies, Södertörn University, Huddinge, Sweden; 9 Department of Organismic and Evolutionary Biology and Museum of Comparative Zoology, Harvard University, Cambridge, MA, United States of America; 10 Gothenburg Centre for Advanced Studies in Science and Technology, Chalmers University of Technology, Göteborg, Sweden

**Keywords:** Sexual selection, Sexual conflict, Mate choice, Polyandry, Speciation, Sensory bias, Signal honesty, Sperm competition, Cryptic female choice, Epigenetics

## Abstract

In recent years, the field of sexual selection has exploded, with advances in theoretical and empirical research complementing each other in exciting ways. This perspective piece is the product of a “stock-taking” workshop on sexual selection and sexual conflict. Our aim is to identify and deliberate on outstanding questions and to stimulate discussion rather than provide a comprehensive overview of the entire field. These questions are organized into four thematic sections we deem essential to the field. First we focus on the evolution of mate choice and mating systems. Variation in mate quality can generate both competition and choice in the opposite sex, with implications for the evolution of mating systems. Limitations on mate choice may dictate the importance of direct vs. indirect benefits in mating decisions and consequently, mating systems, especially with regard to polyandry. Second, we focus on how sender and receiver mechanisms shape signal design. Mediation of honest signal content likely depends on integration of temporally variable social and physiological costs that are challenging to measure. We view the neuroethology of sensory and cognitive receiver biases as the main key to signal form and the ‘aesthetic sense’ proposed by Darwin. Since a receiver bias is sufficient to both initiate and drive ornament or armament exaggeration, without a genetically correlated or even coevolving receiver, this may be the appropriate ‘null model’ of sexual selection. Thirdly, we focus on the genetic architecture of sexually selected traits. Despite advances in modern molecular techniques, the number and identity of genes underlying performance, display and secondary sexual traits remains largely unknown. In-depth investigations into the genetic basis of sexual dimorphism in the context of long-term field studies will reveal constraints and trajectories of sexually selected trait evolution. Finally, we focus on sexual selection and conflict as drivers of speciation. Population divergence and speciation are often influenced by an interplay between sexual and natural selection. The extent to which sexual selection promotes or counteracts population divergence may vary depending on the genetic architecture of traits as well as the covariance between mating competition and local adaptation. Additionally, post-copulatory processes, such as selection against heterospecific sperm, may influence the importance of sexual selection in speciation. We propose that efforts to resolve these four themes can catalyze conceptual progress in the field of sexual selection, and we offer potential avenues of research to advance this progress.

## Introduction

A great deal of the biodiversity on this planet, especially the spectacular traits at which humans marvel, are direct or indirect results of sexual selection. Darwin (1871) defined sexual selection as *“the advantage which certain individuals have over other individuals of the same sex and species solely in respect of reproduction”* (reproduction, in this context, meaning mating success). The flowers of an alpine meadow, antelope horns, a dawn chorus of songbirds: all are snapshots of long histories of sexually selected diversification and exaggeration of signals and displays that have or once had effects on mating or fertilization success. For sexually reproducing organisms, intrasexual competition for reproductive opportunities is a powerful selective pressure, not only shaping the extravagant ‘secondary sexual characters’ that Darwin originally set out to explain, but also with obvious potential to reinforce or even trigger speciation and dramatically affect macroevolution and biogeography. To explore biodiversity without an understanding of sexual selection is a bit like laying a jigsaw puzzle upside down. With our current insight that reproduction is the hard currency of natural selection, it may seem strange that the notion of sexual selection required such a massive volume of reasoning and countless examples (Darwin, 1871), and that despite this effort, it remained controversial for so long. Darwin identified two components of sexual selection: contest competition between rivals of the same sex (typically males) and mate choice (typically by females). Female choice in particular attracted criticism, first from Wallace (1895) and later by others (although, ironically, with arguments similar to the often useful ‘good genes’ models of today; (Cronin, 1991; Hoquet & Levandowsky, 2015; Prum, 2012). Even when Fisher (1930) outlined the intuitively plausible runaway process involving a preferred male trait and a preference gene acting in females in his classic monograph, it was rather skeptically reviewed by Huxley (1938a), Huxley (1938b). However, with the exception of Bateman (1948), the subject was largely ignored until the explosion of evolutionary and behavioural ecology in the 1970s, further sparked by the first demonstration of female choice in the wild (Andersson, 1982). Conceptions of ornamental traits as quality advertisements (Williams, 1966; Zahavi, 1975) and how variation in such viability messages can be maintained (notably Hamilton & Zuk, 1982), together with edited volumes like Bateson (1983) and Bradbury & Andersson (1987), generated questions and research programs for decades to come.

The theoretical genetic modelling of Fisher’s trait-preference coevolution was pioneered by O’Donald (1962), Fisher’s last Ph.D. student, but runaway dynamics were not fully explored and demonstrated until the landmark models of Lande (1981) and Kirkpatrick (1982). These were advocated as the ‘Lande-Kirkpatrick null model’ of sexual selection by mate choice by Prum (2010), partly as a reaction to decades of focus on indicator models and direct or indirect benefits of mate choice (reviewed by Kempenaers, 2007; Kokko et al., 2003; Mead & Arnold, 2004). This and the neglect of the social competition that is the essence of sexual selection (Darwin, 1871; West-Eberhard, 1979), have been called ‘sexual selection amnesia’ by West-Eberhard (2014).

Darwin emphasized adaptations arising from what is now termed pre-copulatory sexual selection, i.e., competition for matings. Since 1970 it has become accepted that sexual selection can continue after mating (post-copulatory sexual selection; (see Birkhead, 2010), and much work has been completed on its two components analogous to Darwin’s male-male competition (i.e., sperm competition Parker, 1970; Parker & Pizzari, 2010) and female choice (i.e., sperm selection or cryptic female choice; Eberhard, 1996; Firman et al., 2017; Thornhill, 1983). Further, it is increasingly appreciated that the process of sexual selection is associated with, and frequently exacerbates, sexual conflict (Table 1), i.e., cases where male and female fitness interests cannot be simultaneously satisfied (Parker, 1979; Trivers, 1972). Thus, after a long period of quiescence since its inception in 1871, the past 40 years have seen an upsurge of interest in sexual selection with the rise of new theory, modern computer technology, molecular biology and techniques in comparative analysis having fueled extensive developments.

**Table 1:** Glossary.

**Anisogamy**	The within-species occurrence of gametes of two different sizes, which results in two sexes, males and females. Females produce the larger and males the smaller gametes.
**Bateman Gradient**	The slope of the linear regression of the number of offspring produced by an individual (reproductive success, or ‘fertility’) on the number of its reproductive partners (mating success). This represents the multiplicative component of the gradient of precopulatory sexual selection acting on a trait. It is named after the seminal study of Bateman (1948), which used fruit flies, *D. melanogaster*, to suggest that the relationship between fertility and mating success is stronger in males, and argued that in an anisogamous population males can have higher potential reproductive rates than females, resulting in more intense intrasexual competition over mating opportunities in males.
**Benefits of mate choice**	**‘Direct’ benefits** of mate choice are ‘non-genetic’ and include resources that will benefit the choosing parent or its offspring, for example access to food, a safe territory, or parental care. **‘Indirect’ benefits** are ‘genetic’ in the sense that by choosing a mate, a parent will secure ‘good’ (viability-related) genes or ‘sexy’ genes (genes for traits that are attractive to the opposite sex) for its offspring, or genes that are compatible to the parent’s own genotype.
**Generalization**	Responsiveness (preference or aversion) to novel stimuli, generated by discrimination learning, and along the dimension(s) of the training stimuli. The resulting **generalization gradients** (e.g., a preference function) can be either a **Peak shift** (peak response to stimuli stronger than the positive training stimulus), or an **Area shift** (peak not shifted, but function asymmetric and biased towards the reinforced direction). Finally, if the gradient does not show a decrease within the interval considered, the preference or aversion can be called **Open-ended** (see e.g., Ghirlanda & Enquist, 2003; Ten Cate & Rowe, 2007 and Fig. 3).
**Genic capture**	Female preferences for costly male traits results in the evolution of a genetic covariance between male condition, dictated by many genes, and a target male trait expression
**Lek paradox**	The problem, commonly relating to female choice of males on leks, of how genetic variation for mate choice can persist despite directional selection for the problem, commonly relating to female choice of males on leks, of how genetic variation for mate choice can persist despite directional selection on traits relevant to choice. Under directional selection, the favoured genes should fixate, so that all individuals of the selected sex should have the gene(s) making them attractive, thus removing the basis for the choice.
**Linkage disequilibrium (LD)**	LD is the non-random association of alleles at different loci. The term often causes confusion and LD may exist without physical linkage or allele frequencies in equilibrium. The speciation-with-gene-flow process is characterized by the build up of LD and genome-wide LD is the footprint of speciation. LD in specific genomic regions reflects the history of selection, gene conversion and other forces that cause gene-frequency evolution.
**Mating preference**	A bias during mate choice which results in a skew towards mating with individuals that express specific phenotypic traits.
**Mating system**	**Monandry** –females mating with one male. **Monogamy** –both sexes mating with one mate. **Monogyny** –males mating with one female. **Polyandry** –females mating with multiple males. **Polygamy (or polygynandry)** –both sexes mating with multiple mates. **Polygyny** –males mating with multiple females.
**Pleiotropy**	One gene affects two or more traits (**genetic pleiotropy**), or one hormone affects two or more traits (**hormonal pleiotropy**).
**Receiver bias**	Used here and by some other authors (Ten Cate & Rowe, 2007) to include all biased responses (preferences or aversions), whether generated by peripheral sensory systems (**sensory bias),** neural processing (**perceptual bias**) or learning or imprinting (**cognitive bias**). Ryan & Cummings (2013) suggest that Sensory and Cognitive bias should be included in Perceptual bias. See Fig. 3
**Receiver psychology**	A phrase coined by Guilford & Dawkins (1991) “to encompass the cognitive mechanisms in signal receivers that process incoming information and could potentially influence signal evolution” (Rowe, 2013).
**Recombination**	The production of offspring with different combination of alleles at different loci than their parents. Recombination often refers to the exchange of genetic material between homologous chromosomes during meiosis (chromosomal crossover).
**Red Queen**	A theory proposing that organisms must constantly evolve in response to their ever-changing environment. The “Red Queen” analogy is derived from Lewis Carroll’s fantasy novel “Through the Looking-Glass” (1871) where the Red Queen tells Alice that “it takes all the running you can do, to keep in the same place”. The Red Queen theory has been applied to many forms of coevolution among species, for example the antagonistic interactions between parasites and their hosts, and the benefit of sex. In sexual selection theory, Hamilton & Zuk (1982) proposed that sexual ornaments signal the bearer’s resistance to parasites, which is a “Red Queen” model assuming a female preference for good genes. The “Red Queen” logic can also be applied to explain female preferences for rare or dissimilar alleles at immune genes that give a broader allelic repertoire and better pathogen resistance in the offspring, as argued here (the “Promiscuous Red Queen” hypothesis, see Fig. 2.)
**Segregation**	Pairs of alleles segregate (separate) into different gametes during meiosis. This is referred to as Mendel’s law of segregation.
**Sensory drive**	A model proposed by Endler (1992) which encompasses evolutionary interactions between the (abiotic and biotic) environment, sensory system and courtship signals, taking into account pre-existing bias and sensory exploitation. Sensory and signalling systems coevolve under the constraints of the environment which hence influence the evolutionary trajectory in a predictable direction (Cummings & Endler, 2018; Endler, 1992).
**Sexual cascade**	The set of sequential evolutionary transitions in sexual strategy of eukaryote organisms, each transition under appropriate conditions giving rise to the selective forces that generate the next. Some taxa remain ‘frozen’ at a given stage without further change. The cascade begins with isogamous syngamy in unicells. Development towards multicellularity favours anisogamy and generates a unity sex ratio. In early, sedentary marine organisms with broadcast spawning, sexual selection is restricted to sperm competition and sperm selection. Development of mobility permits diversion of expenditure on sperm into ‘female-targeting’ (moving to and release of sperm adjacent to spawning females), which may ultimately facilitate internal fertilization and the many forms of pre-copulatory sexual selection documented by Darwin (1871).
**Sexual conflict**	A situation in which the fitness of a male and a female cannot be both maximized separately and simultaneously, by the same trait or reproductive decision. This can arise as social conflict between prospective sexual partners, when a reproductive decision (e.g., whether to mate with each other or not) is adaptive for one individual but detrimental to the other. This conflict is often mediated by sex-limited traits and can give rise to sexually antagonistic patterns of intersexual coevolution in which the antagonistic effect of alleles at some loci is counteracted by the effect of alleles at other loci (*inter-locus*). Another form of ‘conflict’ can arise when there is a divergence in the male and female phenotypic optima, and gene expression is not sex limited. In this case a locus can segregate for different alleles which may have sexually antagonistic effects when expressed in males and females, i.e., an allele that is beneficial when expressed in females may be detrimental when expressed in males and *vice versa* (*intra-locus*).
**Sexual selection**	Selection that depends on the advantage which certain individuals have over other individuals of the same sex and species, in exclusive relation to mating and fertilization (Andersson, 1994; Darwin, 1871).

## Survey Methodology

The enthusiastic resurgence of sexual selection theory in the 1970s and ‘80s stimulated a Dahlem Conference which sought to identify emerging directions (Bradbury & Andersson, 1987) and the intensity of interest in the field has continued unabated. The recent workshop on sexual selection and sexual conflict held at Chalmers University/University of Gothenburg (“Origins of Biodiversity Workshop: Sexual selection and Sexual Conflict”, April 2017) aimed a renewed ‘stock-taking’ on diverse aspects of the subject. Our goal is not to review the entire field, or even subfields, of sexual selection and sexual conflict (e.g., Andersson, 1994; Arnqvist & Rowe, 2005; Birkhead & Møller, 1998; Cummings & Endler, 2018; Eberhard, 1996; Hare & Simmons, 2018; Jones & Ratterman, 2009; Kuijper, Pen & Weissing, 2012; Rosenthal, 2017), but rather to pose a series of open questions emerging from the workshop, naturally colored by our various interests, expertise and empirical systems. The questions we pose delimit broad themes within sexual selection and conflict, answers to which we consider of critical importance to the advancement of the field as a whole. The subsections were either written independently or co-written before being compiled into four research themes within sexual selection (as per Andersson, 1994): (1) the evolution of mate choice and mating systems, (2) sender and receiver mechanisms shaping signal design and evolution, (3) the genetic architecture of sexual selection, and (4) sexual selection and sexual conflict as drivers, or obstacles, of speciation. We hope that these lines of questioning will encourage discussion and offer non-specialists an insight into this ever-expanding area of evolutionary biology.

### 1. Evolution of mate choice and mating systems

Anisogamy, the size difference between male and female gametes that results from the formation of two sexes, is generally accepted as a primary force behind broad patterns of male-male competition over mating opportunities and female-driven mate choice (e.g., Janicke et al., 2016; Schärer, Rowe & Arnqvist, 2012). Over the last decade, there has been a revived focus on anisogamy (Table 1) and its evolutionary consequences (e.g., Janicke et al., 2016; Lehtonen & Kokko, 2011; Lehtonen, Parker & Schärer, 2016; Parker, 2014; Schärer, Rowe & Arnqvist, 2012). The ‘sexual cascade’ (Table 1), a successive sequence of events that has occurred during the long-term evolution of sexual strategy (Parker, 2014; Parker & Pizzari, 2015), provides a null expectation for competitiveness and choosiness in many taxa. Socio-ecological conditions can, however, arise that favor deviations from ancestral behavioural adaptations. Thus, despite the evolutionary irreversibility of anisogamy (Parker, 1983), patterns such as male-mate choice and female–female competition over mates do arise and overwrite the ancestral influence of anisogamy. Much of this is well captured by operational sex ratio theory (Emlen & Oring, 1977;Clutton-Brock & Parker, 1992; reviewed in Kvarnemo & Simmons, 2013), explaining often seen variation in competitiveness and choosiness, also on short time scales (e.g., Forsgren et al., 2004). Indeed, sex-specific investment in competition, mate choice, parental care, and sexual dimorphism vary dramatically across the animal kingdom (Ahnesjö & Bussière, 2016; Janicke et al., 2016), and this variation deserves our attention and interest.

This shift in research interest is reflected by a number of reviews within the last decade demonstrating the prevalence of female competition and male choice (Edward & Chapman, 2011; Hare & Simmons, 2018; Rosvall, 2011; Schlupp, 2018; Stockley & Bro-Jørgensen, 2011). Importantly, these behaviors are not restricted to species where there is an *a priori* expectation of sex-role ‘reversal’, because male-mate choice can co-occur with female mate choice, and similarly, both sexes can show intra-sexual competition for mating opportunities. When both sexes vary in their quality as mates, selection can generate mating competition and selective mate choice in either sex (Owens, Burke & Thompson, 1994; Owens & Thompson, 1994; Parker, 1983). It is therefore critical to our understanding of sexual selection that we do not let preconceived ideas about sex roles limit our predictions and study designs.

Below we examine a few general topics related to mate choice and mating systems (Table 1). How and why organisms choose their partners may hinge on direct contributions to the quality of a reproductive bout or indirect genetic benefits. We discuss how details of pre- and post-copulatory processes can affect sexual selection, and how genetic benefits that derive from mating with a particular individual might be important in the context of both pathogens and inbreeding. Finally, we point out benefits of studying broadcast spawning, as this form of reproduction excludes pre-copulatory sexual selection. Future research into the relative contributions of direct vs. indirect benefits should take into account mating systems, temporal limitations placed on mate choice, and other selection pressures.

#### 1a. Direct and indirect benefits of mate choice—implications for mating systems and sexual selection

Mate choice can be time consuming, risky and might even result in individuals that are too choosy not succeeding in finding a mate. We therefore expect individuals to gain important benefits from mate choice to cover these costs. Mate choice can evolve through the pursuit of both direct and indirect benefits (‘benefits of mate choice’, Table 1) and can take the form of either pre- or post-copulatory selection (Edward & Chapman, 2011; Jennions & Petrie, 2000). Whereas mate choice for direct benefits primarily occurs before mating, mate choice driven by indirect benefits can continue after mating, and may be particularly important if the genetic quality of potential mates cannot be determined prior to mating. Post-copulatory mate choice therefore requires mating with multiple mates.

In some taxa, such as migrating passerine birds, pair formation and therefore pre-mating mate choice occurs under severe time stress (e.g., Alatalo, Carlson & Lundberg, 1988; Bensch & Hasselquist, 1992; Dale & Slagsvold, 1996). This likely puts a premium on mate choice for direct benefits such as territory quality and social partner condition. A hasty assessment of indirect attributes such as ‘good’, ‘sexy’ or compatible genes (explained under ‘benefits of mate choice’, Table 1), can then be corrected afterwards by mating with additional (extra-pair) partners. This ‘correction’ can either take the form of trading-up, that is, mating with an extra partner only if the additional partner’s genetic quality is better than that of the current social partner(s), or it can be achieved after mating with multiple partners via post-copulatory processes such as sperm competition and cryptic mate choice (Jennions & Petrie, 1997; Jennions & Petrie, 2000).

Genetic compatibility within mated pairs is a key aspect of mate choice that is attributed to selection for indirect benefits. Post-copulatory mate choice for complementary genes involved in immune function has been shown in fish, mammals and lizards (Olsson et al., 2003; Penn, 2002; Penn & Potts, 1999, see also ‘*Is extrapair mating a “Promiscuous Red Queen”?’ and ‘Inbreeding avoidance: when markers matter*’). In procellariform birds, high olfactory bulb-to-brain ratios co-occur with long-term genetic monogamy (Bried, Pontier & Jouventin, 2003; Zelano & Edwards, 2002; Zelenitsky et al., 2011), and genetic compatibility based mate choice (Strandh et al., 2012). Might olfaction be causally linked to the evolution of mating systems? If there is such a link, taxa with relatively larger olfactory bulbs would be expected to be better at accurate mate choice for genetic compatibility prior to mating, possibly promoting long term genetic monogamy in such taxa (Colegrave, Kotiaho & Tomkins, 2002). For example, a recent study shows low levels of extra-pair paternity and male-mediated mate choice based on Major Histocompatibility Complex (MHC) loci in a largely monogamous seabird (Hoover et al., 2018). On the other hand, mating systems other than monogamy (e.g., polygyny in lek-breeding species) may also promote olfaction based mate choice.

More research is needed to identify sexually selected traits contributing to direct benefits. When an individual can increase its mating success by offering direct benefits, then the traits that contribute to such benefits (e.g., being fecund, in good condition, able to secure and defend a fine territory, having good parenting skills) may be subject to mate selection. Given a genetic basis of a trait it can also respond to selection. Importantly, this means that many traits that are traditionally seen as products of natural selection are likely to also be affected by sexual selection, and hence pushed away from their naturally selected optima. That parental care can be under sexual selection is already well established (Kvarnemo, 2010; Lindström & St. Mary, 2008), but a broader appreciation of other dually selected traits is likely to improve our understanding of trait evolution.

#### 1b. The influence of polyandry on sexual selection and sexual conflict

The level of polyandry of a population will likely reflect the outcome of interactions between male- and female-driven strategies. Whereas male strategies are often assumed to drive and females to resist polyandry, some degree of polyandry can be adaptive and actively promoted by females. Importantly, polyandry is likely to have drastic effects on the operation of sexual selection on males. The key implication is that polyandry creates a new source of variation in male reproductive success in the form of variation in paternity share arising from multiple matings by females and male-male competition over access to fertilization.

The resulting two episodes of postcopulatory sexual selection (sperm competition and cryptic female choice; see above) add considerable complexity to the architecture of variation in male fertilization success (Webster et al., 1995), and consequently to the operation of sexual selection. Recent work has demonstrated that, contrary to previous expectations (e.g., Møller, 1998), polyandry can severely limit variation in fertilization success among males, which weakens precopulatory sexual selection on male mating success. This process can often drastically reduce the total opportunity for sexual selection on males, relegating it primarily to postcopulatory episodes (Collet et al., 2012; Jones et al., 2001; Shuster & Wade, 2003). One important consequence of this effect is that polyandry acts to reduce the difference between male and female Bateman gradients (Parker & Birkhead, 2013; ‘Bateman gradient’, Table 1).

Theory on the interaction between female strategies of sperm selection and male strategies of sperm allocation needs expansion and further development. For instance, early observations of increased proportional paternity in less as compared to more closely related males (Olsson et al., 1996) were found robust when controlling for effects such as unfertilized eggs and parental inbreeding-induced early offspring mortality (Olsson et al., 1999; Olsson et al., 1997). However, male ejaculation economics could also be influenced by detection (e.g., based on olfactory cues) of relatedness with the female and competing rivals (Olsson et al., 2004) as could female sperm choice *per se*, a supposition supported by male–female relatedness interactions on a male’s probability of paternity (Olsson et al., 1996). Female strategies may range from mechanical manipulation of ejaculates to biochemical selection for sperm in the female tract and at the ovum surface (Firman et al., 2017). Patterns of cryptic female choice may thus influence male sperm allocation to matings (Ball & Parker, 2003). Male strategies involve numerous trade-offs, e.g., between pre-mating expenditures such as mate searching, and post-mating expenditures on sperm allocation, paternity guarding and paternal investment. The nature of precopulatory male-male competition (e.g., contest *vs.* scramble) also affects expenditure on pre- and post-mating male adaptations (Parker & Birkhead, 2013). So far, while some evidence exists for a trade-off between pre- and post-mating expenditures (Kvarnemo & Simmons, 2013), it appears that the nature of precopulatory male-male competition is complex, and may be influenced by covariation between the scramble-contest axis and the level of polyandry (sperm competition) (reviewed in Parker, 2016).

**Figure 1:**
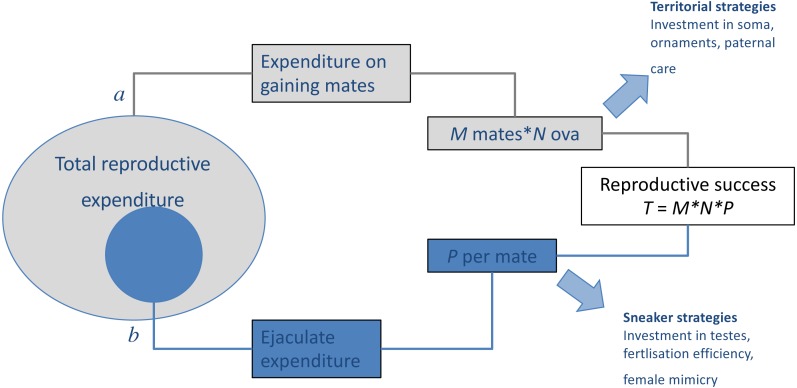
Results of trade-offs between pre- and post-copulatory investment in polyandrous species. A male’s reproductive success (i.e., the total number of offspring produced, *T*) is determined by: (A) the number of females with whom he mates successfully (mating success, *M*) and their fecundity (i.e., average number of ova produced, *N*), and (B) the proportion of these that he fertilises (*P*). When reproductive resources are limited, males face a trade-off between investment in precopulatory (A) and postcopulatory competition (B). Under some conditions, such trade-off can have alternative optima for different male types, setting the scene for alternative mating tactics, in which a discrete phenotype, which invests preferentially in attracting and monopolising females (e.g., territorial), co-exists and competes with phenotypes, which invest preferentially in sperm competition (e.g., sneaker or satellite). Adapted from Parker (1998).

When there is negative covariance between male (precopulatory) mating success and (postcopulatory) paternity share, such trade-offs may play a considerable role in the evolution and maintenance of alternative mating tactics (Fig. 1). As more fine-grained data on mating behaviour become available, detailed studies of the distribution of polyandry within populations and its ramifications on sexual selection can be developed, investigating for example how mating success of individual males correlate with the polyandry of their sexual partners (McDonald & Pizzari, 2014; McDonald et al., 2013; McDonald & Pizzari, 2016; Sih, Hanser & McHugh, 2009). This parameter represents the extent to which precopulatory sexual selection on male mating success (male ‘Bateman gradient’) can be strengthened or weakened by the distribution of polyandry in a population (McDonald & Pizzari, 2016).

Finally, while polyandry was originally assumed to exacerbate sexual conflict, there is increasing appreciation that polyandry may have a more nuanced effect, by relaxing conflict over some precopulatory decisions (e.g., mating rates Parker & Birkhead, 2013), while creating conflict over postcopulatory reproductive decisions, such as female selection of sperm or paternal care.

#### 1c. Is extrapair mating a “Promiscuous Red Queen”?

Birds provide a particularly interesting study system for genetic polyandry because they often copulate with partners outside the socially monogamous pair bond. Since the advent of molecular parentage testing tools in the 1980s, hundreds of paternity studies in birds have revealed that extrapair paternity is common, though the proportion of offspring sired by extrapair males is quite variable across and even within species (Griffith, Owens & Thuman, 2002; Westneat & Sherman, 1997). Nevertheless, the question of why and how this variation in extrapair mating is maintained, especially among closely related species with similar phenotypes, ecology and life history, is still unresolved.

The first generation of hypotheses attempting to explain patterns of paternity share in birds focused on how the opportunity for extrapair copulations may vary with breeding density (Birkhead, Atkin & Moller, 1987; Westneat, Sherman & Morton, 1990) and breeding synchrony (Stutchbury & Morton, 1995; Westneat, Sherman & Morton, 1990). Although these factors might explain some of the variation within species, they do not explain the broader picture of variation in extrapair paternity rates across species (Bennett & Owens, 2002; Westneat & Sherman, 1997). Consequently, over the last two decades, several attempts have been made to correlate extrapair paternity rates with various other variables linked to ecology and life history variation. Some evidence suggests that high extrapair paternity rates are associated with fast life histories, reduced paternal care, sexual dichromatism, social monogamy (as opposed to polygyny; ‘mating systems’, Table 1), seasonal migration and temperate breeding (reviewed in Arnold & Owens, 2002; Bennett & Owens, 2002; Hasselquist & Sherman, 2001; Spottiswoode & Moller, 2004). However, there are two major problems with these ‘second generation’ explanations; they explain rather small proportions of the total variance among species, and causal mechanisms for how they influence extrapair paternity are difficult to infer.

Similar conclusions were reached in a recent study restricted to Passerides songbirds (Lifjeld et al., 2019) where species with higher extrapair paternity rates show stronger sexual dichromatism, are more migratory, and have reduced male care at the initial stages of the breeding cycle (nest-building and incubation). However, effect sizes were small and the direction of causality obscure. For example, the relationship with sexual dichromatism was largely due to changes in female, not male, coloration, which might be explained by plumage adaptations in females to promiscuous behaviour (i.e., more crypsis). Similarly, males may respond to high extrapair paternity rates by allocating more effort to extrapair mating than to parental care at the early stages of the nesting cycle when more females are available for extrapair copulation (Westneat, Sherman & Morton, 1990). Hence, patterns of association may reflect consequences rather than causes of variation in extrapair paternity. These results imply a sobering conclusion that neither factors associated with social opportunities, ecology and life history variation, nor male secondary sexual traits, can explain the large variation in genetic polyandry documented among bird species in general or among songbirds in particular. Additionally, rates of extrapair paternity carry a rather weak phylogenetic signal (Lifjeld et al., 2019), which suggests that the behaviour is an evolutionarily labile trait that responds rapidly to changing selection pressures.

How then can the diversity in avian genetic mating systems be explained? Extrapair mating is an arena for sexual conflict where females might be better positioned to win in terms of controlling the process of internal fertilization, despite the higher value of winning for males (Lifjeld & Robertson, 1992). Petrie & Kempenaers (1998) argued that variation in this behavior can only be understood by considering the benefits, costs and constraints to female choice. Their paper is a timely reminder, since some more recent studies seem to dismiss an adaptive role for female extrapair mating due to a lack of empirical evidence for female genetic benefits (e.g., Arnqvist & Kirkpatrick, 2005; Forstmeier et al., 2014). Clearly, if female extrapair mating is adaptive, the benefits must either be direct (fertility insurance) or indirect (‘good’, ‘sexy’ or compatible genes), since females seem to obtain nothing but sperm through extrapair copulation. An implication of this assertion is that female genetic benefits could be small or non-existent in species with low rates of extrapair paternity, and that evidence for female genetic benefits should primarily be sought among species with extensive female extrapair mating. There is indeed evidence for genetic benefits, such as a higher cell-mediated immune response, (Arct et al., 2013; Fossoy, Johnsen & Lifjeld, 2008; Garvin et al., 2006; Johnsen et al., 2000), increased heterozygosity (Foerster et al., 2003; Fossoy, Johnsen & Lifjeld, 2008; Stapleton et al., 2007; Tarvin et al., 2005) and enhanced reproductive success for offspring sired by extrapair males (Foerster et al., 2003; Gerlach et al., 2012) in passerine species with high extrapair paternity rates.

There is evidence to indicate a key role for genes involved in immune function. Passerine birds have higher rates of extrapair paternity than other clades of birds (Griffith, Owens & Thuman, 2002). They also have much more polymorphic and duplicated MHC genes (Hess & Edwards, 2002; Minias et al., 2018; O’Connor et al., 2016; Westerdahl, 2007; O’Connor et al., 2019), which play an important role in the adaptive immune system. These patterns could be causally linked. A study on eight species from the passerine sister families Muscicapidae and Turdidae found a positive correlation between extrapair paternity rates and sequence diversity at the peptide-binding sites of MHC class II molecules (Gohli et al., 2013). In one of these species with high extrapair mating, the bluethroat *Luscinia svecica,* individuals can have up to 56 different alleles and thus a minimum of 28 duplicated loci (Rekdal et al., 2018). Gene duplications ensure a high within-individual allelic repertoire and can be favoured under high pathogen pressure (Bentkowski & Radwan, 2019; Minias et al., 2018). Mate choice for resistant mates or mates that enhances the pathogen resistance in offspring will reinforce the natural selection for gene duplications. The positive correlation between extrapair mating and MHC diversity and duplication would therefore suggest that species that face strong pathogen-mediated selection evolve an extrapair mating strategy for immunogenetic benefits.

**Figure 2:**
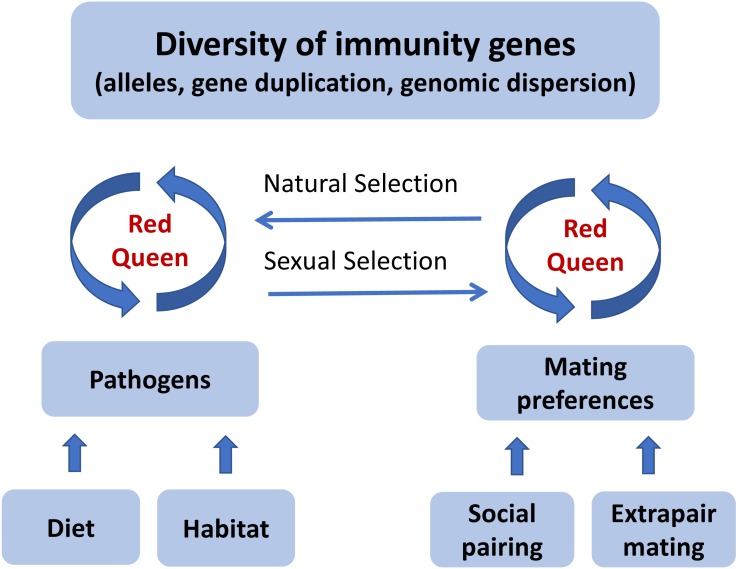
A graphical illustration of the “Promiscuous Red Queen” hypothesis for the evolution of immune gene diversity and variation in female promiscuity. The diversity of immune genes in a population is shaped along two selection pathways, both subject to the Red Queen dynamics of host-parasite coevolutionary cycles (see text box). The first one, which is relevant for all species, is natural selection caused directly by pathogens resulting in differential survival of alleles. The strength of selection is determined by the abundance, diversity and virulence of pathogens in the environment, primarily exposed through diet and habitat-specific variables. The second pathway, sexual selection, kicks in when random mating (with respect to immune genes) is an inferior strategy compared to a mating preference for certain alleles. For species that form pair bonds, mating preferences can theoretically be exerted both in the pairing process and in subsequent extrapair matings, and can either target specific alleles (good genes) or alleles that make a good match to the female’s own genotype (compatible genes). Pathogen-mediated selection can therefore act directly on organisms through a natural selection pathway, and indirectly through a sexual selection pathway, under a “Red Queen” scenario. When social mate choice is largely driven by non-genetic resource benefits and is random with respect to genes, genetic preferences can be exerted in extrapair mate choice. Females can thereby get the best (resources and genes) out of two separate choice situations. When social monogamy constrains female choice of genes, extrapair mating will evolve. The stronger the genetic benefits through pathogen-mediated selection on offspring fitness, the more effort females should devote to extrapair mating. When beneficial alleles increase in frequency and pathogens become less harmful, extrapair mating becomes less important. The “Promiscuous Red Queen” model is thus a possible explanation to the variation in extrapair mating systems observed among species and populations, especially in passerine birds.

In a ‘Red Queen’ (Table 1) coevolutionary dynamic between pathogens and host immunity, the strength of pathogen-mediated selection may fluctuate within a species over time, and also vary among species with similar ecology and distribution at any point in time. If social mate choice does not provide enough options for females to choose the better genes, extrapair mating might evolve as an alternative mating strategy. Once most individuals have acquired an effective allelic repertoire to fight off pathogens, or social mate choice offers sufficient options, the benefit of female extrapair mating will be reduced and the mating system will revert towards sexual monogamy. This ‘Promiscuous Red Queen’ hypothesis (Fig. 2) can therefore explain why divergence in extrapair mating systems evolves rapidly among closely related species.

Genotyping of hypervariable and highly duplicated genes like the passerine MHC with next generation sequencing methods holds a great potential for testing predictions of the Promiscuous Red Queen hypothesis in species with extensive extrapair mating and highly diversified immune genes (Lighten, Van Oosterhout & Bentzen, 2014; O’Connor et al., 2016; Sebastian et al., 2016; reviewed by O’Connor et al., 2019). Many sets of samples utilized previously for paternity studies should be readily available for testing of MHC diversity.

Female extrapair mate choice for immune genes can result in different non-random combinations of parental alleles. Females might prefer specific beneficial alleles (good genes) or alleles that make a good match to her own alleles (compatible genes). What constitutes a favourable allelic match should be investigated by analyzing the fitness of individuals with different allelic repertoires (Milinski, 2006). If maximum allelic diversity (in terms of number or sequence diversity of alleles) yields the highest fitness, females should choose males with dissimilar alleles (Strandh et al., 2012). If an intermediate allelic diversity is optimal, as too many alleles can lead to autoimmunity, then females should choose a mate that gives an intermediate allelic diversity in the offspring (reviewed in Milinski, 2006). Regardless of what the optimal allelic diversity for individuals could be, the model predicts that extrapair offspring should have an allelic diversity closer to the population optimum than that of within-pair offspring. If the optimum lies close to the population mean, then observed choices may not differ from each other or from a random model in mean values, only in variances. Even if the mate choice optimum lies close to the population mean and there is stabilising selection (reduced variance) around this optimum in an ecological time frame, MHC diversity can still increase over evolutionary time if the optimum moves (Estes & Arnold, 2007).

A further challenge will be to reveal a possible mechanism for the mating preference; either there could be pre-copulatory cues for a behavioral discrimination among males, or cryptic female sperm selection mechanisms in the oviduct or at the ovum (Firman et al., 2017). A recent study reported that the chemical composition of preen wax reflects similarity at MHC II genotypes in a songbird (Slade et al., 2016), which opens up the possibility for pre-copulatory mate choice based on olfactory cues in passerine birds, as previously documented in seabirds (e.g., (Strandh et al., 2012) (Direct and indirect benefits of mate choice—implications for mating systems and sexual selection).

#### 1d. Inbreeding and mate choice—when are relatives preferred?

Inbreeding affects fitness negatively in a wide range of taxa (Crnokrak & Roff, 1999; Keller et al., 1994; Keller & Waller, 2002), with an increase in genome-wide homozygosity in the offspring of related parents. Resulting effects on fitness can arise through partial dominance or overdominance; the result of either being the promotion of inbreeding avoidance mechanisms (Charlesworth & Charlesworth, 1987; Marr, Keller & Arcese, 2002). However, inbreeding may not systematically result in selection for inbreeding avoidance, and it is challenging to predict when an organism avoids, tolerates or even prioritizes consanguineous matings (Szulkin et al., 2013). Building on well-developed theory that underpins similar phenomena in organisms such as plants, where selfing is common, a consideration of both the costs of inbreeding avoidance and benefits of inclusive fitness is necessary. If inbreeding costs are sufficiently low, both sexes can be selected to inbreed (Kokko & Ots, 2006; Parker, 1979; Parker, 2006) as a means to promote gains in inclusive fitness among related individuals. This can be achieved through extrapair copulations, although such mating decisions may come at the cost of a decrease in the fitness of extrapair relative to within-pair young (Lehtonen & Kokko, 2011). However, although an increase in inclusive fitness was suggested as an explanation for matings among related individuals as long as four decades ago (Parker, 1979), it has remained widely ignored by animal ecologists. More recent advances in evolutionary genetic theory have restored interest in questions related to inbreeding biology (Kokko & Ots, 2006), and empirical data show refined mate choice based on female inbreeding status. In burying beetles, only females that are inbred themselves, with greater risk of a genetic compromise by inbred partners, choose outbred males (Pilakouta & Smiseth, 2017).

Future work should address the relationship between sexual selection and inbreeding in wild animal populations (see ‘*Inbreeding avoidance: when markers matter*’). Recent software developments, such as Rhh (Alho, Valimaki & Merila, 2010), have proven very useful to investigate large data sets focusing on the effects of inbreeding on the process of sexual selection and other components of fitness (Bebbington et al., 2017; Forstmeier et al., 2012). Furthermore, progress in genomic and theoretical investigations of inbreeding (Hedrick & Garcia-Dorado, 2016) and sexual selection (Anthes et al., 2017) provide a thorough foundation for future work on aspects of sexual selection and inbreeding biology in the wild. In the next section we take a closer look at how individuals may avoid inbreeding.

#### 1e. Inbreeding avoidance: when markers matter

Inbreeding avoidance can occur through polyandry (Bretman, Wedell & Tregenza, 2004; Firman & Simmons, 2008; Foerster et al., 2003; Olsson et al., 1996; Simmons et al., 2006; Tregenza & Wedell, 2002), dispersal (Bollinger, Harper & Barrett, 1993; Greenwood, 1980; Pusey, 1987), and kin recognition (Gerlach & Lysiak, 2006; Hoffman et al., 2007). In the latter, MHC haplotypes have been proposed as a cue associated with kin discrimination, due to potential correlations between the degree of shared MHC alleles and genome-wide relatedness (Brown & Eklund, 1994; Penn & Potts, 1999; Potts & Wakeland, 1993). Individuals that mate with MHC dissimilar partners are then expected to avoid potential fitness costs associated with inbreeding, while optimizing (Kalbe et al., 2009; Madsen & Ujvari, 2006; Reusch et al., 2001) or maximizing offspring MHC heterozygosity, via heterozygote advantage (Doherty & Zinkernagel, 1975) or negative frequency dependence (Hedrick, 2002; Milinski, 2006; Slade & McCallum, 1992).

MHC genes encode glycoproteins that bind pathogen-derived peptide fragments on cell surfaces, and thus play an important role in the immune system (Janeway et al., 2001; see ‘*Is extrapair mating a “Promiscuous Red Queen”?*’). Therefore, two selective forces may underlie MHC-based mate discrimination, inbreeding avoidance and enhanced immunocompetence. The function of MHC in mate choice and the importance of disentangling these two fitness-related phenomena has been demonstrated in wild Atlantic salmon (*Salmo salar*) (Landry et al., 2001), and in the Swedish sand lizard (*Lacerta agilis*) (Olsson et al., 2003). Specifically, mated salmon pairs showed greater dissimilarity at their functional MHC class II β proteins than expected under random mating, but did not exert mate discrimination according to genetic relatedness or inbreeding avoidance.

Although MHC loci may still act as a cue for kinship in some systems (reviewed by Penn & Potts, 1999; Spurgin & Richardson, 2010), MHC similarity between mated pairs should be interpreted with a degree of caution. It is essential to distinguish between degree of kinship and MHC similarity, and avoid generalization with regards to the genetic mechanisms underlying differential reproductive investment in vertebrates. In other words, a sound scientific approach in studies of disassortative mating patterns relies on an adequate choice of genetic marker.

#### 1f. Research on broadcast-spawning invertebrates can advance the field of sexual selection

While Darwin (1871) dismissed the ‘lowest classes’ from sexual selection, it is now appreciated that sexual selection can indeed operate in such taxa, albeit in different ways (Levitan, 1998). There is every reason to suppose that even in sedentary broadcast spawners, sexual selection can affect gamete traits (Evans et al., 2012; Evans & Sherman, 2013), gonads and even life history traits (Parker et al., 2018). For example, eggs exposed to experimental sperm mixtures can discriminate between sperm from different male genotypes (Palumbi, 1999), and sperm move preferentially towards more genetically compatible ova (Evans et al., 2012). Gonadosomatic indices of conspecific males and females can vary considerably as a result of sperm competition and sperm limitation levels, relative costs to the sexes of gonad tissue and gamete production, and the trade-off between growth and reproduction (Parker et al., 2018).

One of the benefits of studying broadcast spawning invertebrates in the context of sexual selection is that they represent an early stage in the sexual cascade (Parker, 2014; Parker & Pizzari, 2015; Table 1), capturing a phase before the evolution of enhanced mobility and behavioural complexity, which, as Darwin realized, was essential for the evolution of adaptations through pre-copulatory sexual selection. Since it is often difficult to separate pre-and post-copulatory components of sexual selection, sedentary broadcast spawning invertebrates present a unique opportunity to study the type of adaptation that can arise through sexual selection and sexual conflict at the gametic level, eliminating pre-copulatory considerations. A question that arises is why these taxa have remained ‘frozen’ at a sedentary level, without selective forces favouring increased mobility and female targeted gamete release, since traces of such behaviour are seen in ‘pseudo-copulation’ in some echinoderms (Keesing et al., 2011) and pairing behaviour in certain cnidarians (Tiemann et al., 2009).

### 2. Sender and receiver mechanisms shaping signal design

The proximate physiological and neurological mechanisms for production, emission and perception of signals are essential keys to both adaptive and non-adaptive aspects of sexual communication. In particular, the design and evolutionary trajectories of signals are shaped by both *content* (e.g., accuracy and honesty of quality advertisements), and *efficacy* (e.g., sensory ecology and receiver psychology). In the sections below, we discuss developments and challenges in these two areas.

First, the mediation of signal honesty in many study systems likely depends on a dynamic and complex integration of social and physiological costs, which may be both spatially and temporally variable. It can be a formidable empirical challenge to measure the “right” parameters at the right time, but for detailed understanding of honest signaling, this is the way forward.

Second, and especially relevant to the biodiversity theme of our workshop, we address the increasingly appreciated impact of receiver biases (sensory, perceptual or cognitive) on both design and diversification of sexually selected traits (see e.g., Cummings & Endler, 2018; Ryan & Cummings, 2013; Ten Cate & Rowe, 2007). Here also lie great empirical challenges, for example to objectively identify and quantify the relevant dimensions of signal traits, signaling conditions and sensory tuning, to experimentally demonstrate receiver biases, and, in appropriate cases, to phylogenetically reconstruct the origins and contingencies of these traits.

#### 2a. Mediation of signal honesty in a dynamic framework: integration of social and physiological costs

Several models of sexual selection predict that signal traits are honest indicators of individual quality (Andersson, 1986; Folstad & Karter, 1992; Grafen, 1990; Hamilton & Zuk, 1982; Zahavi, 1975). An implicit prediction of these models is a consistency in the physiological mediation of honesty, that is, the costs associated with the trait should be fairly constant over time. This assumption is likely unrealistic given that physiological condition can change drastically, even over short periods of time (e.g., changes in physiology due to illness or a variable environmental context), while many signal traits are produced once and are fairly static. Further, the expression of signal traits is relative in the sense that the same signal can be viewed as more or less exaggerated, depending upon the social context, such as the signal intensity of conspecifics in the population. The mechanisms that allow morphological signal traits to convey relevant information within a changeable social context is an interesting puzzle, especially in cases where traits are developed and then fixed for a set period of time during which reproductive transactions take place, such as horns and many aspects of plumage that are developed annually. For dynamic traits that can be modulated in real-time, such as song rate or acrobatic courtship display, the problem becomes a bit less complicated because signalers can behaviorally adjust to changing physiological conditions and social context.

There is recent appreciation that even static signal traits have an active rather than constant relationship with physiology and behavior, which likely has important implications for determining how these signals remain coordinated with behavior as social contexts change (e.g., Merrill et al., 2014; Safran et al., 2008; Tibbetts, 2014; Vitousek, Zonana & Safran, 2014). Still, questions remain about if and how these interactions maintain the transmission of honest information to conspecifics.

A cornerstone of both physiological and social cost models of honest signaling is that signal costs are less steep for high-condition compared to low-condition individuals, which creates variation in optimal signal expression (Grafen, 1990). Social challenge of signal expression is relatively robust to this assumption (for a review see Webster, Ligon & Leighton, 2018), but necessarily reliant on frequency of challenge and either potential or realized social costs. Physiological costs can also vary conditionally, for example, both testosterone-induced immunosuppression and glucocorticoid-related ectoparasite load differ based on the quality of the signaler in blue tits (Roberts & Peters, 2009) and sand lizards respectively (Lindsay et al., 2016). Webster and colleagues (2018) argue that physiological costs, although subject to intensive scrutiny in the last few decades, may be a less evolutionarily stable mechanism for honest signal mediation than social costs. Where selection for social punishment of cheaters should increase as the benefits of social status become higher, selection should favor a decoupling between costly physiological processes and trait expression, such as through upregulation of target sensitivity to hormonal stimulus. However, the limited empirical support for physiological cost models of honest signal mediation (i.e., immunocompetence handicap hypothesis; Roberts, Buchanan & Evans, 2004) may instead reflect the challenges of detecting these costs.

These challenges include the following. (1) The pleiotropic actions (Table 1) of key biomarkers of physiological state, such as pro- and antioxidants, testosterone, and glucocorticoids, can have contradictory effects on different body systems, requiring measurement of a broad panel of physiological costs. For example, simultaneous and opposing relationships have been detected between hormone titre and endo- vs. ectoparasite load (Fuxjager et al., 2011; Lindsay et al., 2016). (2) Time-lags between when biomarkers are elevated and when they exert their influence can obscure detection of costs and necessitate repeat sampling and a knowledge of multiple interacting physiological systems. For example, a direct link between oxidative stress and telomere length has been difficult to establish (Boonekamp et al., 2017), but when this relationship was examined across multiple sampling periods, it became clear that telomere length near the end of life is strongly predicted by measurements of oxidative stress experience earlier in life whereas simultaneously measured oxidative stress was unrelated (Olsson et al., 2018). (3) Physiological production costs are presumably accrued during a brief time-window of ontogeny, often distinct from the period in which the signal is utilized in socio-sexual interactions. This necessitates researchers to have a deep knowledge of how and when signals are formed and requires application of appropriate experimental procedures during these critical time frames.

The degree to which social enforcement vs. physiological costs mediate signal honesty likely varies with social context (gregariousness, presence of dominance hierarchies, population density) and it is clear that social costs can have physiological outcomes and vice versa. For example, testosterone stimulates aggressive behavior, and social aggression itself can increase testosterone further (“challenge hypothesis”, Wingfield et al., 1990). Such aggressive social engagement can simultaneously influence production of glucocorticoids (Creel, 2001; Creel et al., 2013), which, in turn, can impact investment in reproductive behaviors and testosterone production (Sapolsky, Romero & Munck, 2000). Both hormones have been causally and correlationally linked to signal expression in multiple systems (Cote et al., 2010; Cox, Zilberman & John-Alder, 2008; Fernald, 1976; Leary & Knapp, 2014; Lendvai et al., 2013; Lindsay et al., 2016; Lindsay, Webster & Schwabl, 2011; Mougeot et al., 2004; Peters et al., 2000) and the relationship between signal and hormone titre itself can be bidirectional (Laubach et al., 2013; Safran et al., 2008; Tibbetts, Crocker & Huang, 2016). If an individual is in poorer condition than when the signal was produced (and any production costs accrued), secondary physiological costs associated with carrying and defending an elaborate signal may accumulate. An emerging mismatch then, between the intensity of the signal and the behavior and apparent health of the signaler, allows the receiver to assess true condition (i.e., “integrative incongruence hypothesis”, Tibbetts, 2014), despite the fact that the signal itself may remain seasonally static.

Ideas for future questioning and caveats to this type of research have been addressed elsewhere (Tibbetts, 2014; Vitousek, Zonana & Safran, 2014; Webster, Ligon & Leighton, 2018). Studies that include observations of trait and behavior combinations with explicit full-factorial tests that adjust signal intensity, behavior, and measure consecutive and simultaneous social and physiological costs are needed. Such research should be paired with examination of long-term fitness consequences of potential costs.

#### 2b. Receiver mechanisms and biases that shape signal design

*“Sensory biases may cause elaboration in the absence of the Fisherian process…and more reasonably be the null hypothesis and primitive model on which to build other components of sexual selection”* (Price et al., 1987).

Conspicuous flowers and fruits attracting pollinators and dispersers; aposematic and warning signals; social and sexual threat displays; nature is full of signals that have been exaggerated without hitch-hiking with a genetically correlated preference, but simply by exploiting a biased detection, preference or aversion in the intended receiver. Such receiver biases can be sensory, perceptual or cognitive (Ryan & Cummings, 2013), hardwired or learning-based (Ten Cate & Rowe, 2007), adaptive or neutral, or even maladaptive if compensated by benefits in another context in which the bias is adaptive and perhaps originated.

Like all communication signals, sexual displays can be deconstructed into two defining properties: information content, and efficacy (Andersson, 2000; Guilford & Dawkins, 1991). Traditionally, models of sexual selection were concerned with the adaptive significance of female choice and whether the information content of male ornaments conveyed direct or indirect (genetic) fitness consequences (Andersson, 1994; see also ‘*Direct and indirect benefits of mate choice implications for mating systems and sexual selection*’, above). In contrast to such ‘sender-precursor models’ (see Bradbury & Vehrencamp, 2011) of signal evolution, ‘receiver-precursor models’ shift focus to efficacy aspects such as signal conditions (background, attenuation) and receiver properties, by exploring how signal design may originate and be exaggerated to exploit sensory or cognitive receiver biases (collectively termed ‘perceptual biases’ by Ryan & Cummings (2013). Empirically this was triggered by classic studies of preferences and biases that phylogenetically seemed to pre-date the visual or acoustic signal trait (Basolo, 1990; Ryan et al., 1990). Additionally, exploitation of pre-existing biases has been suggested as a common origin of sexual signal evolution (Arnqvist, 2006). Yet, despite the obviously crucial importance that receiver properties must have for signal design and evolution (Guilford & Dawkins, 1991; Guilford & Dawkins, 1993; Jansson & Enquist, 2005), studies of sexual signal evolution have, with some notable exceptions (Arak & Enquist, 1993; Enquist & Arak, 1993), largely neglected receiver psychology (Table 1), and studies of receiver psychology have rarely interpreted results in an evolutionary context.

While most studies of receiver biases in sexual selection have focused on mate choice, the application of receiver precursor models to agonistic (threat) signaling systems presents a very different context. Firstly, agonistic signals have the potential to be emancipated from the constraints of direct linkage to male quality; some mechanism must maintain signal honesty, which may be achieved through socially mediated costs. Thus, agonistic signals may be more evolutionarily labile than epigamic signals, potentially allowing for higher rates of change in signal form. Secondly, the time scale for signal information to manifest can be much shorter for agonistic signals; a male can test the honesty of another male’s signal directly. This interaction also sets the stage for a learning-based receiver bias, essentially a discrimination task analogous to those shown to generate ‘generalization’ in the psychology literature (Ghirlanda & Enquist, 2003; Ten Cate & Rowe, 2007; Table 1). Therefore, agonistic signaling systems may be ideal candidates for investigating the influence of receiver biases on signal design and exaggeration. Indeed, recent studies have revealed ongoing selection by receivers on agonistic signal design, compatible with patterns of convergent evolution in the direction of a receiver preference (Ninnes & Andersson, 2014; Ninnes et al., 2015; Ninnes, Webb & Andersson, 2017).

**Figure 3:**
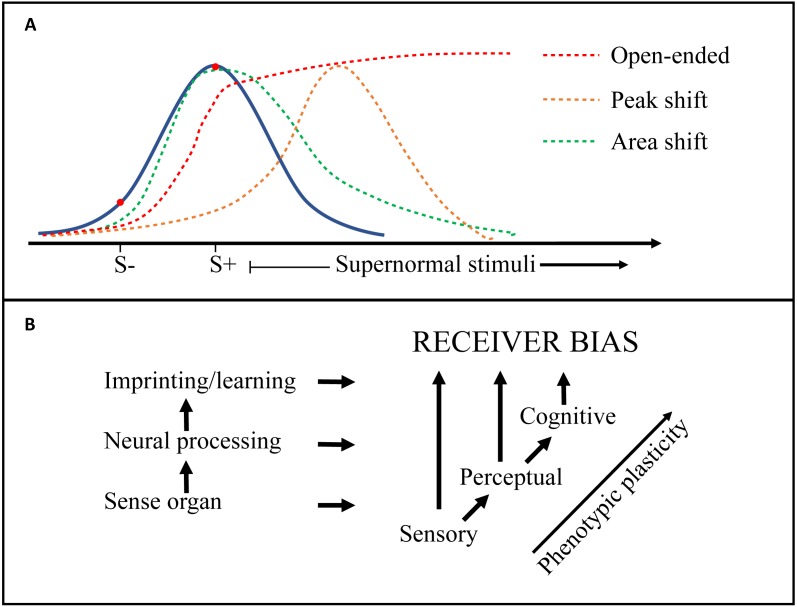
Generalization gradients and origins of receiver bias. (A) Receiver biases exert directional selection on a signal trait (e.g., tail length in birds) and may create heightened responsiveness to supernormal stimuli. The blue curve depicts responsiveness by an unbiased receiver. Peak shift (orange line), area shift (green line), and open-ended (red line) ‘generalization gradients’ (see Table 1: Glossary) are generated by discrimination learning, which here is illustrated by a negative (S −) and a positive (S +) training stimulus. (B) Other receiver biases can also derive directly from a peripheral sensory bias (e.g., in the retina), or from the higher level ‘Perceptual’ processing of the sensory input (e.g., visual cortex). The general increase in phenotypic plasticity from peripheral to higher level neural processing is indicated.

One of the primary challenges for research into this field is to tidy up the definitions and terminology used in regard to receiver psychology. Whereas the environmental constraints and selective forces on both senders and receivers are well covered and structured in the Sensory Drive model (Cummings & Endler, 2018; Endler & Basolo, 1998), there is some confusion regarding the terms used to describe the neurological mechanisms of receiver biases (e.g., sensory, perceptual, cognitive), as well as the implications for signal selection (e.g., supernormal stimulus, generalization, peak-shift) (Endler & Basolo, 1998; Ghirlanda & Enquist, 2003; Ryan & Cummings, 2013; Ten Cate & Rowe, 2007). Figure 3 is an attempt to distinguish some of these terms and how they relate to each other, but many questions remain. For example, are ‘pre-existing biases’ inherent hard-wired preferences, or a function of the psychology of discrimination tasks (i.e., generalization; Ghirlanda & Enquist, 2003; Ten Cate & Rowe, 2007)? Future work should seek to integrate conceptual frameworks from biology and psychology to help elucidate mechanistic processes. For example, an examination of ‘pre-existing biases’ in signal design should include methods standard to the field of psychology such as the generation of response gradients by testing responses at multiple points on a signal dimension. Second, is the impact of receiver psychology on sexual signal design, through selection on signal efficacy, underappreciated? ‘Virtual evolution’ experiments have suggested that receiver biases similar to empirically demonstrated generalization gradients (Jansson & Enquist, 2003), are sufficient to drive signal exaggeration (Jansson & Enquist, 2005). This aligns with for example the consistent and pre-existing receiver biases found in closely related widowbirds and bishops (*Euplectes* spp), displaying varying degrees of signal exaggeration (Ninnes & Andersson, 2014; Ninnes et al., 2015; Ninnes, Webb & Andersson, 2017; Pryke & Andersson, 2002). Echoing previous researchers including Endler (1992), Ryan & Cummings (2013) and West-Eberhard (2014), we suggest that intensified attention to the origins, mechanisms and response gradients of receiver biases will bring us closer to the neuroethology of signal selection and the design and diversity of sexual signals. In ‘*Mate choice and ecological speciation*’ we also discuss some of the implications of evolving receiver preferences on speciation.

Lastly, the notions of ‘aesthetic preferences’ and ‘beauty’, used in both Darwins and Fishers writings on female choice, have been treated as objective biological traits (Prum, 2017; Renoult, Bovet & Raymond, 2016), leading to heated debate (e.g., Borgia & Ball, 2018; Patricelli, Hebets & Mendelson, 2018). Renoult & Mendelson (2019) argue that aesthetic preferences represent neurobiologically efficient and thereby adaptive cognitive processing, strongly resembling the ‘inevitable signal recognition biases’ suggested by the artificial neural network models of Enquist & Arak (1993). Recent controversy has been instigated by Prum (2012) and Prum (2017) who argues not only that the Fisherian process should be the null model of the evolution of mating preferences, but that any evolved cognitive bias is the ‘aesthetic sense’ while the exaggerated signal properties define ‘beauty’. These assertions received several critical responses (e.g., Borgia & Ball, 2018; Patricelli, Hebets & Mendelson, 2018), also from quarters that agree with Prum that “mate choice for indicators is often assumed as an explanation for the evolution of elaborate displays without sufficient consideration of other processes” (Patricelli, Hebets & Mendelson, 2018).

In our own view, perceptual and cognitive biases are likely to be key components of the ‘aesthetic sense’ that Darwin (1871) attributed to choosy females (see also Renoult, 2016; Renoult, Bovet & Raymond, 2016). Moreover, since perceptual biases may drive ornament or armament exaggeration without involving any sender-receiver genetic covariance or the Fisher process (Price et al., 1987), it may be a simpler, and more testable, ‘null model’ of signal selection in general, and as regards sexual selection, it would apply to both mate choice and contest competition.

### 3. Genetic architecture of sexual selection

Understanding the genetic architecture of sexual selection, and thus evolvability and constraints on sexually selected traits, is a long-term goal of the field and one where substantial progress has been made in recent years. Notable examples include advances in our understanding of the genetic basis of stripes in cichlid fish (Kratochwil et al., 2018), QTL loci underlying song in Hawaiian crickets (Ellison & Shaw, 2013) and other insects (Gleason et al., 2016), morphological traits known to be targets of sexual selection in birds (Hansson et al., 2018), and genes involved with conversion of red carotenoid pigments in birds (Lopes et al., 2016; Mundy et al., 2016). There is an increasing number of studies that demonstrate convincing heritability of key sexually selected traits, like copulatory organs or chemical signaling, and there are several examples demonstrating the evolutionary *consequences* of sexual selection, such as incompatibilities between species (Rose, Brand & Wilkinson, 2014). However, the great progress in identifying genes associated with morphological, coloration and signaling traits known to be under sexual selection has not been accompanied by similar demonstrations of predicted evolutionary signatures in many such genes. Specifically, no example exists, to our knowledge, in which the genetic basis of a sexually selected trait has been shown to evolve rapidly in response to recent or ongoing sexual selection, either experimentally, in the lab, or in nature.

In contrast, rapid evolution is easier to detect in reproductive genes such as accessory gland and reproductive proteins (Finn & Civetta, 2010; Hurle et al., 2007; Wyckoff, Wang & Wu, 2000) and many gene systems associated with interactions between sperm and egg as well as copulatory proteins have been identified in *Drosophila*. Proteomic approaches are adding detail to our understanding of the complex chemical cocktails exchanged during mating in flies, primates and other groups (Claw et al., 2018; Gotoh et al., 2018; Wilburn et al., 2018). The predicted rapid evolution of genes involved in co-evolutionary interactions between the sexes, and between hosts and parasites, has been demonstrated repeatedly. For example, immune genes that may serve as ‘good genes’ such as MHC genes (see ‘*Is extrapair mating a “Promiscuous Red Queen”?*) undergo a type of cycling characterized by rapid evolution (Eizaguirre et al., 2012). A greater understanding of the evolutionary dynamics of genes underlying signaling and performance traits therefore stands as a major gap in our field.

#### 3a. What genes underlie variation in performance?

A goal of contemporary research in the field of sexual selection is the identification of candidate loci for performance. Detailed and often time-intensive field studies of sexual selection are required to identify the phenotypes associated with display or mating success. When combined with modern sequencing techniques, these types of data make it possible to contrast the expression levels or genotypes of the successful individuals with the unsuccessful, revealing key loci underlying measures of performance. Although in principle straightforward, almost no published studies have used such a protocol (but see Johnston et al., 2013). While sequencing on a large scale can still be cost prohibitive, perhaps more importantly, the type of detailed behavioral observations producing reliable individual data on complex parameters like “mating success” are expensive in terms of investment in time and in effort. Field studies on the great snipe (*Gallinago media*) illustrate the latter point (Höglund et al., 2017). To obtain reliable sample sizes, the field work has been conducted over many years under sometimes harsh field conditions and the data is subject to problems inherent to all multi-season datasets, such as observer, site and year effects.

Importantly, genotype effects on mating success may be context dependent, as appears to be the case in great snipes. The effect of candidate SNPs (Single Nucleotide Polymorphism) on great snipe mating success depended on whether birds were infected with avian malaria, as revealed by significant interaction terms among infection status and genotype in a few loci (Höglund et al., 2017). Genomic studies of sexual selection are emerging (see ‘*Genomic properties of speciation through sexual selection*’), and more such are required to make general conclusions. For this to be possible, long term studies with careful observations and detailed knowledge of natural history combined with genomic data is the only remedy.

#### 3b. Genic capture and ongoing sexual selection: how many genes are enough?

A classic question in sexual selection theory is to what extent the evolution of secondary sexual traits is constrained by the exhaustion of genetic variation resulting from the process of selection itself (‘the lek paradox’; Andersson, 1994; Kirkpatrick, 1982; Table 1). In some cases trait expression is dictated by allelic variation at a single locus, whereas in others trait expression is polygenically determined. If genetic variation limits exaggeration of secondary sex traits, this effect should decrease with the number of loci dictating trait development. For example, mating with close relatives contributes to loss of genetic variation and thus, inbreeding opposes sustained sexual selection and secondary sex trait evolution (Keller & Waller, 2002). Empirical research in the fields of sexual selection and evolutionary genetics are inconsistent in terms of the generality of these fundamental processes.

Much discussion has been directed toward the investigation of genetic architecture of multilocus signaling traits with the underlying idea that strongly condition-dependent traits capture all the genetic variance in condition (‘genic capture’, Rowe & Houle, 1996; Tomkins et al., 2004; Table 1). Since many loci provide a large target for mutations, genetic variation could persist over time despite strong directional selection. Work on genic capture has until recently been largely theoretical, because the genotypes of few phenotypic traits are usually unknown in natural populations. An example demonstrates how genetic variation for a strongly polymorphic secondary sex trait, horn type in Soay sheep, is maintained by a trade-off between natural and sexual selection in a single gene (*RXFP2*, Johnston et al., 2013). Horn shape is under strong sexual selection in males, but not in females, so another hypothesis, intra-locus sexual antagonism (see ‘*How do genome processes impact sexual selection and sexual conflict?*’) could also be rejected (Johnston et al., 2013). Work on field caught *Drosophila,* however*,* showed that even with substantial genetic variance in a secondary sex trait, cuticular hydrocarbons, the vast majority of this variation was not closely associated with the direction of sexual selection (Hine, Chenoweth & Blows, 2004). Despite condition-dependence of traits, genetic variation underlying trait expression can be depleted by sexual selection in the wild and thus genic capture did not offer a resolution to the lek paradox in this system. In an interesting empirical example of genic capture, chemical mutagenesis of the male guppy (*Poecilia reticulata*) germline negatively affected courtship displays but not colouration, indicating that the former is a large mutational target (Herdegen & Radwan, 2015). Such mutagenic approaches, when complemented with whole-genome sequencing to verify affected loci, offer a robust approach to study mutational targets, but are limited in their applicability to sexual selection on polygenic traits in the wild. Although the presence of only a few genes can be adequate for evolution of secondary sexual characters to proceed in some systems, multiple and variable genes may not be enough to sustain character evolution in other systems.

#### 3c. How do genome processes impact sexual selection and sexual conflict?

Although much empirical research related to sexual selection has been conducted extensively at the organismal level, little progress has been made in identifying the genomic mechanisms responsible for various sexually selected traits (but see Johnston et al., 2011). Because sexual dimorphism is often the evolutionary outcome of sex-specific selective patterns such as sexual selection, understanding the molecular basis of sexually dimorphic traits is key to understanding evolution by sexual selection. Whereas sex-biased gene expression has been documented in various tissues in many taxa, demonstrating dimorphism at the molecular level (e.g., Leder et al., 2010; Mank et al., 2010; Zhang et al., 2007), it is unclear in many cases whether sex-biased genes are actually antagonistic or if they are a result of current or past antagonistic effects (Parsch & Ellegren, 2013). Additionally, although there are numerous theoretical papers connecting the evolution of sex chromosomes, sex-biased expression and sexual antagonism (e.g., Kirkpatrick & Guerrero, 2014; Mank, Hosken & Wedell, 2014; Parsch & Ellegren, 2013), it has been difficult to test hypotheses in the wild (but see empirical advances by Hollis et al., 2014; and review by Mank, 2017). Much of the difficulty in identifying the genomic bases of sexually selected traits is due to our limited understanding of the genome. It is increasingly feasible to gather DNA, mRNA and protein sequence data, yet understanding genomic and proteomic modifications, such as epigenetics or protein phosphorylation, and the details of interactions among molecules is also necessary to understand the final phenotype.

It has become widely accepted that regulatory variation is the likely source for much of the observed phenotypic variation among and within species (e.g., Carroll, 2008), and regulatory differences have been implicated as a mechanism resulting in sexual dimorphism (Williams et al., 2008). If one considers the concept of intra-locus conflict, where males and females exhibit different fitness optima at a genomic locus, conflict may be resolved by differential regulation of that gene in males and females without dramatic changes in the genome. As suggested by Rice (1984), the sex chromosomes may be hotspots for sexually antagonistic genes, but they also provide a potential mechanism for resolving both intra- and inter-locus conflict through the maintenance of sex-specific alleles. In effect these alleles must be largely regulatory, since there is little unique information on the sex chromosomes in many known systems, and recent work shows that noncoding regulatory sequences alone are sufficient to drive sex reversal in mice (Gonen et al., 2018). Consistent with this idea, replacement of Y chromosomes between species of flies results in genome-wide changes in gene expression, mediated by regulatory factors encoded on the Y chromosome (Branco, Hartl & Lemos, 2013; Sackton et al., 2011). Additionally, organisms without sex chromosomes still exhibit sex differences, most basically in gonad formation and physiology, but also in behaviour. Thus differential regulation leading to sexual dimorphism must be achieved through regulatory cascades that in some cases can be initiated by one or few genes, or even by the environment (Bachtrog et al., 2014).

Another question is which ontogenetic or polyphonic stage to sample individuals in order to understand the genetic basis of a sexually selected trait. Much of the obvious morphological and behavioral differences between the sexes are studied in sexually mature organisms, yet the molecular bases for many of these differences, particularly morphology or coloration, are likely due to differential expression initiated early in development before the trait becomes obvious (Hubbard, Jenkins & Safran, 2015). This is the case with sexually dimorphic abdominal pigmentation in *D. melanogaster* (e.g., Williams et al., 2008), and most studies that identify differences in gene expression between species or ontogenetic stages are in fact identifying regulatory differences (Mallarino et al., 2016). This early development of dimorphism makes it difficult to associate the phenotypic differences observed in adult organisms with specific DNA differences or mRNA expression that may underlie the trait. Studies examining the molecular basis of sexually selected signals in birds often focus on the seasonal elaboration of traits such as plumage color in an effort to identify relevant genes (Lopes et al., 2016; Mundy et al., 2016). New epigenetic techniques, such as ATAC-seq, can identify regions of the genome with open chromatin, unwound from nucleosomes and available for binding by transcription factors, and promise to identify new ways in which the genome can be differentially modulated between the sexes without requiring differences in DNA sequence (Buenrostro et al., 2015).

Molecular pleiotropy and the physical location and recombination environment of a gene may constrain its evolvability and ease of study (see ‘pleiotropy’, Table 1). For example, many proteins form complexes with other proteins or bind to DNA or RNA in order to carry out their function. These interactions limit the mutations that a given gene can accumulate before it is non-functional (Papakostas et al., 2014). Additionally, many genes are pleiotropic and may influence several, even quite different biological processes by being expressed at different times, in different tissues or by forming complexes with different protein partners. Linkage and recombination can also affect the evolvability of genes (Table 1). Genes that are in close proximity on a chromosome will likely be inherited together, thus linked allelic combinations of these genes will tend to be inherited together. In some cases linked loci can even become fixed, as when a chromosomal inversion occurs, creating a ‘supergene’ with diverse effects on the breeding phenotype (Kupper et al., 2016; Lamichhaney et al., 2016; Tuttle et al., 2016). Clearly, a better understanding of genome processes as well as how genes interact and are expressed in both sexes will aid in the understanding of sexually selected traits and sexual antagonistic genes.

### 4. Sexual selection and sexual conflict as drivers, or obstacles, of speciation

Sexual selection is an important evolutionary force in the context of speciation (e.g., Kraaijeveld, Kraaijeveld-Smit & Maan, 2011; Panhuis et al., 2001; Ritchie, 2007; Schaefer & Ruxton, 2015). Traditionally, research in this field has focused on the role of sexual selection during early phases of population divergence, because divergence in display traits and preferences can quickly cause pre-zygotic isolation (Coyne & Orr, 2004). This focus is not surprising given the huge variation we observe in sexually selected traits among relatively newly formed, closely related species. However, sexual selection through mate choice is unlikely to lead to speciation by itself (Ritchie, 2007; Servedio & Burger, 2014), an argument that has resulted in a growing interest in understanding sexual selection in the broader context of ecological speciation (Martin & Mendelson, 2014; Scordato et al., 2014).

There is also a growing awareness that cryptic forms of female choice, i.e., post-mating/post-spawning processes resulting in conspecific sperm precedence, may be important sources of reproductive isolation (Howard, 1999; Palumbi, 2009; Swanson & Vacquier, 2002; Van Doorn, Luttikhuizen & Weissing, 2001). In addition, male-male competition (Qvarnström, Vallin & Rudh, 2012; Tinghitella et al., 2018) and sexual conflict (reviewed by Parker, 2006) are becoming increasingly recognized as important mechanisms of speciation. Below, we discuss these novel lines of progress in our understanding of the role of sexual selection in speciation. Additionally, we provide some suggestions for use of genomic methods in testing current controversies in the field.

#### 4a. Mate choice and ecological speciation

The vast majority of theoretical models evaluating the role of sexual selection in speciation are based on *Fisherian* processes of sexual selection (Lande, 1981). Why has the interest in benefit-driven mate choice been so slow in being transferred from research on sexual selection within populations to research on the role of sexual selection in speciation processes? At least one identified potential “problem” with benefit-driven mate choice in the context of speciation is associated with the unidirectional nature of choice. Disruptive selection is considered to be a prerequisite for population divergence under gene flow but disruptive selection on benefit-driven mate choice is generally not expected. Moreover, while differences in natural selection experienced by geographically separated populations may quickly lead to divergence in male display traits (Maan & Seehausen, 2011), mating preferences (Table 1) may not change in a similar manner. For example, when a long bird tail signals some type of quality and males have evolved shorter tails in one population due to high local costs (e.g., strong predation pressures), females from that population would not be expected to prefer males with relatively short tails. Females from this population of short-tailed males should instead be expected to prefer to mate with males from long-tailed populations whenever they have a chance to do so.

There are several possible solutions to this “problem”. First, female mate preferences may actually experience corresponding natural selection pressures as male display traits. Segami Marzal et al. (2017) found that cryptic female poison frogs experienced elevated predation risk when associating with an aposematic partner. Hence, predation may act directly on female choice favoring the evolution of preferences for less conspicuous males. Second, female mate preferences may be exposed to other environment-specific natural selection pressures that target their sensory system, resulting in population specific mate choice targets (‘sensory drive’, Table 1, Boughman, 2002; Endler, 1992). In short, if a sensory trait, for example vision, is locally adapted and also involved in finding mates or assessing their quality, this functional linkage may result in divergence in male display traits (Boughman, 2002; see also ‘*Receiver mechanisms and biases that shape signal design*’). Moreover, Schluter & Price (1993) suggested that several male traits may reveal the same type of benefits but the perception of these traits may differ between environments resulting in different traits being the prime targets for benefit-driven mate choice in different environments. Empirical evidence suggest multiple effects of female sensory traits causing divergence in male courtship traits (Boughman, 2001; Boughman, 2002; Fuller & Noa, 2010; Seehausen et al., 2008). Finally, a third possible solution is that mate preferences remain the same but assortative mating between populations that are adapted to different environments is still possible (Kopp et al., 2018). For example, immigrant males that lack genes underlying local adaptation are unable to develop large ornaments, such as bright coloration, enabling females to discriminate against them (Van Doorn, Edelaar & Weissing, 2009). Males that are well adapted to the local environment will therefore be more attractive to females, and offer direct benefits (e.g., territory quality) or genes that are related to local adaptation (reviewed in Safran et al., 2013). Thus, under certain prerequisites, genes that contribute to adaptation will spread in the population through both natural and sexual selection. In some systems, however, rare immigrants to a population appear to achieve enhanced survival and lower parasite loads compared to residents (Bolnick & Stutz, 2017). In ‘*How does sexual conflict impact speciation processes?*’ below, we discuss how genomic approaches can be used to test the prerequisites for sexual and natural selection to jointly promote speciation.

#### 4b. Cryptic female choice and post-copulatory reproductive isolation

Choice mechanisms directly based on conspecific sperm traits rather than species-specific secondary sexual traits are known from external fertilizers, like abalones, sea urchins and oysters (Vacquier & Swanson, 2011) and fish (Yeates et al., 2013). Post-copulatory reproductive barriers are much less known in internal fertilizers, at least partly because of the difficulty of studying what goes on within the female reproductive tract (Birkhead & Brillard, 2007). However, there is increasing evidence for such “cryptic” mechanisms of female choice, where heterospecific sperm is discriminated against also in internally fertilizing animals, like insects (Coyne & Orr, 2004) and non-passerine birds (Birkhead & Brillard, 2007).

Although pre-copulatory mate choice based on plumage and song traits is well known in passerine birds, little attention has been paid to possible post-copulatory reproductive barriers. Passerine sperm morphology is known to evolve rapidly (e.g., Hogner et al., 2013) and the rate of evolution is positively related to the risk of sperm competition (Rowe et al., 2015). One emerging question is therefore whether sperm divergence could be causally involved in reproductive isolation between incipient species pairs with sperm competition, through differential fertilization success of conspecific over heterospecific sperm. At the mechanistic level, this could work via co-evolution between sperm length and sperm storage tubule length (Briskie & Montgomerie, 1992). In other words, sperm of a heterospecific male might be selected against because they are not the right size to fit in the sperm storage tubules. Alternatively, reproductive proteins in seminal- and ovarian fluid, which are known to evolve rapidly in other taxa (Turner & Hoekstra, 2008, but see Rowe et al., 2018), may be the key molecules involved in post-copulatory selection mechanisms also in passerines.

A recent study of two sympatric *Ficedula* flycatchers, suggests that female pied flycatchers (*F. hypoleuca*) that are constrained to pair with heterospecific males, are more prone to perform extra-pair copulations with conspecific males and able to exert cryptic choice in favour of their sperm, thereby reducing the risk of producing unfit hybrid offspring (Cramer et al., 2016a). By *in vitro* testing of sperm velocity from males of each of the two species against cloacal fluid collected from females of both species, the authors found an asymmetric pattern: sperm from collared flycatcher (*F. albicollis*) males experienced a higher velocity reduction in pied flycatcher female fluid than in collared flycatcher fluid, but not vice versa. Furthermore, this effect was strongest for pied flycatcher females with a high likelihood of previous exposure to sperm of collared flycatcher males. Such effects were not seen in studies of four other, non-hybridizing passerine species pairs, with a range of divergences in genetic distance and sperm morphology (Cramer et al., 2014; Cramer et al., 2016b), suggesting that selection against hybridization may have favored the evolution of this cryptic barrier in flycatchers.

Future studies, targeting the molecular mechanisms underlying sperm performance within conspecific and heterospecific female reproductive environments, will shed novel light on the type of selection acting at this cryptic level of female choice and the relative importance of pre- and post-copulatory mate choice in speciation processes.

#### 4c. Male–male competition and niche segregation

When males compete over females or resources needed to attract females, they often bias their aggression towards the most common male phenotype in the population (Qvarnström, Vallin & Rudh, 2012; Tinghitella et al., 2018). This means that both the invasion of, for example, new color morphs and stable polymorphism within populations become much more likely than in cases when mate choice acts as the main mechanism of sexual selection (reviewed in Qvarnström, Vallin & Rudh, 2012; Tinghitella et al., 2018). One may argue that such negative frequency-dependent selection driven by male aggression could promote divergence in e.g., color morphs with little divergence in niche use. In agreement with this line of reasoning, Seehausen & Schluter (2004) found that sibling species of cichlid fishes in Lake Victoria were ecologically similar but markedly different in coloration. Closely related species of cichlids with similar color were also less likely to occupy the same habitat patches (Seehausen & Schluter, 2004). Should we then expect the diversifying aspects of male-male competition to be unrelated to ecological speciation?

There are at least three main reasons to expect that divergence in sexually selected traits used in male contest competition may often be associated with divergence in niche use. First, dominance hierarchies are often asymmetric between color morphs and population divergence in traits used in combat (e.g., horns, large bodies) is often directly associated with dominance strategies and thereby access to other resources than females (Forsgren, Kvarnemo & Lindström, 1996; Qvarnström, Vallin & Rudh, 2012). We therefore predict population divergence in sexually selected traits used in male-male competition and population divergence in niche use to often be associated. Second, at secondary contact between young species, selection against heterospecific aggression may contribute to increased niche segregation. Ongoing habitat segregation was for example observed in a recently formed hybrid zone between collared and pied flycatchers on the Swedish island, Öland. An asymmetry in male contest competition ability over nesting sites needed to attract females resulted in male pied flycatchers being displaced from deciduous forests patches into less preferred mixed forest habitats (Vallin et al., 2012). As a consequence of this habitat segregation, the access to resources used to feed nestlings declined dramatically in breeding territories used by pied flycatchers but the risk of hybridizing with collared flycatchers also declined (Rybinski et al., 2016). Thus, habitat segregation not only led to reduced aggressive interactions between the two flycatcher species, but also to reproductive isolation. Third, environmental effects on the efficiency of different signaling traits may not only affect which traits become targets of female choice by being relatively more detectable or reliable (Schluter & Price, 1993) but also which traits become targets of male competition. Lackey & Boughman (2013) compared limnetic and benthic species of threespine stickleback fish across different habitats. They found that mixed habitats favored two trait combinations and thereby likely divergence and reproductive isolation while homogenous open habitats favored only one trait combination and thereby likely hindered trait divergence and reproductive isolation (Lackey & Boughman, 2013).

#### 4d. How does sexual conflict impact speciation processes?

In contrast to sexual selection, less research has targeted the consequences of sexual conflict on speciation. Several approaches concur with the notion that sexual conflict will also catalyse speciation, others suggest the reverse (see reviews by Gavrilets, 2014; Parker, 2006). The hypothesis that selection favours restriction of gene flow when hybrids between ecotypes have a fitness disadvantage relies on the tacit assumption that female interests will prevail in mating decisions. However, unless the hybrid disadvantage is sufficiently great, it will be in male interest to mate (Kokko & Ots, 2006; Parker, 1974; Parker, 1979; Waser, Austad & Keane, 1986); a wide parameter zone exists over which sexual conflict applies and in this zone selection on females acts as a force favouring speciation by restricting gene flow, but selection on males acts as a force resisting speciation by promoting gene flow. While some empirical studies suggest that sexual conflict promotes speciation, others do not (Gavrilets, 2014; Plesnar-Bielak et al., 2013).

Extending this argument, Parker & Partridge (1998) suggested that under sexual conflict, ‘female win’ resolutions in given taxa may result in high species numbers and low genetic variation per species, whereas ‘male win’ resolutions may result in taxa with low species numbers and high genetic variation per species. Which solution prevails depends on the value of winning (generally greater for males) and ‘power’, a measure related to the fitness costs of overcoming the current defense by the opposite sex (costs for females of preventing mating may often be less than the costs for males of imposing matings). Similarly, Magurran (1998) proposed that sexual conflict and male interests may be key to explaining the absence of speciation in Trinidadian guppies, *Poecilia reticulata*, where population differentiation is nevertheless high and female choice appears to reinforce divergence. Sneak mating by males is common, and may generate sufficient gene flow to prevent reproductive isolation. Early comparative attempts to establish a link between speciation rate and possible proxies for sexual conflict (sexual size dimorphism, polyandry) in mammals, butterflies and spiders were unsuccessful (Gage et al., 2002), but recent work on shorebirds also gives some support to the notion that male interests (measured in terms polygamy) can act against speciation (D’Urban Jackson et al., 2017).

The role of sexual conflict in speciation certainly deserves further investigation. An interesting complication is that if two subpopulations, A and B, have diverged sufficiently, the fitness consequences to males and females of A and B can become asymmetric, e.g., the relative hybrid disadvantage in (i) male A ×female B matings may differ from that in (ii) female A x male B matings. Additionally, the balance for the sexes between the fitness value of winning (i.e., between mating or not mating) and ‘power’ (the fitness cost of overcoming defences) may differ in these two possible pairings (Parker, 1979; Parker & Partridge, 1998). Such asymmetries could hypothetically lead to a variety of situations: for example, sexual conflict could occur in case (i) but not in case (ii), so that (depending on the ‘value of winning value’/‘power’ balance) selection could favour speciation in one population but not the other, a form of “speciation conflict”.

#### 4e. Genomic properties of speciation through sexual selection

Genomic approaches may help to bridge several important gaps in our current understanding of the role of sexual selection in speciation. Detailed information about the genomics underlying sexually selected phenotypes can be used to test key assumptions of theoretical models on sexual selection (Wilkinson et al., 2015, see also ‘*Genetic architecture of sexual selection*’ above) and then be placed into the context of speciation. Because, as mentioned above, divergent sexual selection alone rarely causes speciation (Ritchie, 2007), one particularly interesting aspect of ‘the context of speciation’ deals with how traits involved in several different aspects of reproductive isolation can remain in linkage disequilibrium under gene flow (Butlin & Smadja, 2018; Coyne & Orr, 2004; Seehausen et al., 2014; Table 1). Hybridization can easily break up crucial trait-combinations through recombination and segregation (Table 1, Felsenstein, 1981). The completion of speciation under gene flow is therefore considered to be more likely when traits involved in reproductive isolation have dual functions (Gavrilets, 2004; Slatkin, 1982; Smadja & Butlin, 2011). The completion of speciation occurs because, when a single trait is under divergent natural selection and also involved in mate choice, the association between these two functions cannot be easily broken by recombination. There are numerous examples of putative multiple effect traits (‘magic’ traits) involved in population divergence, many focusing on the signaling side of sexual selection (Servedio et al., 2011; Smadja & Butlin, 2011). One of the best examples is from *Heliconius* butterflies, where the mimicry pattern also has a signaling function when acquiring mates (Kronforst et al., 2006; Merrill et al., 2011). However, mate preferences can also function as ‘magic’ traits with dual functions. For example, in the context of sensory drive speciation (Table 1) in teleost fishes. In short, if a sensory trait, for example vision, is locally adapted and also involved in finding mates or assessing their quality, this means a functional linkage between niche use and mate choice. Given the difficulties in unravelling the genetic background of especially mate choice, these systems could be good candidates for studies of genetic architecture of mate preferences (Table 1). In *Pundamilia* cichlid fish and *Heliconius* butterflies, where gene flow is evident and multiple effect traits have been invoked to be instrumental in the speciation process, empirical results are consistent with few genes having a major effect on female assortative mating (Haesler & Seehausen, 2005; Kronforst et al., 2006; Merrill et al., 2011; Svensson et al., 2017).

When several different traits contribute to reproductive isolation, linkage disequilibrium among the underlying loci may shelter against the homogenizing effects of gene flow. Barton (1983) introduced the term ‘coupling’ to refer to a process where buildup of linkage disequilibrium between loci under divergent selection promotes speciation (Flaxman et al., 2014). Such coupling occurs because each locus with an effect on reproductive isolation is then not only influenced directly by selection acting on itself but also by indirect selection acting on the other coupled loci leading to stronger overall isolation. Much scientific attention has been directed to possible genetic coupling by physical linkage between isolation loci through proximity on particular chromosomes (e.g., sex chromosomes Qvarnstrom & Bailey, 2009), particular parts of chromosomes with low recombination rates (e.g., centromeres Ortiz-Barrientos, Engelstadter & Rieseberg, 2016) or within recently formed chromosomal rearrangements (Noor et al., 2001). Empirical studies suggest that differentiated loci are indeed enriched in genomic regions with reduced recombination (Wolf & Ellegren, 2017) but such patterns alone need to be interpreted with caution. Genomic studies need to be tightly intertwined with knowledge about phenotypic effects to reveal which differentiated loci that have effects on reproductive isolation as differentiation *per se* does not impose a key function in the speciation process. To achieve this goal several different methods need to be combined. First, ecological and behavioral studied are needed to reveal the function of phenotypic traits and their role in niche use, mating and most importantly their barrier effects - their role in causing reproductive isolation. Second, the genetic variants underlying these traits need to be revealed with genome wide association studies or similar (GWAS, Rockman, 2012). Finally, Butlin & Smadja (2018) recently suggested that more scientific attention also needs to be directed towards the coupling processes themselves and that the term ‘coupling’ should be extended to include any process that generates coincidence of barrier effects. Reaching these three goals is a challenging empirical undertaking but would reveal key information about the speciation process, including the role of sexual selection in driving reproductive isolation.

## Conclusions

Our survey of emerging questions in sexual selection, while necessarily incomplete, shows that the field is on the cusp of a major revolution. In many ways the theoretical framework for the study of sexual selection and sexual conflict is robust, having been refined since the late 1960s. What is needed now are bold empirical attempts to understand the diverse molecular and ecological mechanisms that could modulate the outcomes of sexual selection and sexual conflict.

One obvious frontier of sexual selection resides in increased understanding of the molecular genetic and physiological mechanisms of traits subjected to or contributing to sexual selection and sexual conflict, an understanding that next-generation molecular methods will help achieve. Although interesting in its own right, it is perhaps even more important what these mechanisms imply about the history, constraints and evolvability of traits, allowing several outstanding issues in sexual selection and sexual conflict to be addressed. A molecular understanding of sexually selected traits will help the field discriminate between alternative hypotheses for the maintenance of variability in those traits, for example, whether they have evolved via good genes mechanisms or by more arbitrary or neutral processes (Prum, 2010; Prum, 2017). A good example is the recent elucidation of the genes involved with carotenoid metabolism in birds (Lopes et al., 2016; Mundy et al., 2016; Toews, Hofmeister & Taylor, 2017). With a clear understanding of the genes that process ingested carotenoids, we can gain better estimates of the true costs and constraints on those traits, which in turn can help predict their evolutionary trajectories within and between species.

It would, however, be short-sighted to conclude that molecular mechanisms alone will bring a holistic understanding of sexual selection and conflict. Genetic mechanisms only have meaning when appropriately placed in the context of the natural history and ecological and social constraints that characterize different systems exhibiting sexual selection. Recent examples show how molecular methods achieve their biggest impact when deployed in the context of large-scale ecological and behavioral studies of naturally occurring variation in the wild (e.g., Bosse et al., 2017). And although an understanding of the historical origins of traits, i.e., ancestral constraints and exaptations, is (or should be) the very essence of modern evolutionary biology, there is still a striking lack of ‘tree-thinking’ that would facilitate understanding such constraints in biology in general, and sexual selection in particular (Price, Clapp & Omland, 2011). This trend is particularly true in the study of the many micro- and macroevolutionary consequences of sexual selection (but see Prum, 1997).

Advances at the interface of molecular, ecological, behavioral and theoretical research will require collaborations between experts in divergent areas, a goal that we hope our workshop in Gothenburg has fostered.

## Additional Information and Declarations

### Data Availability

The following information was supplied regarding data availability: This article is a literature review and is not accompanied by data.
